# A simple fixation for detection of endogenous fluorescent reporters in *C. elegans* that nearly eliminates intestinal autofluorescence

**DOI:** 10.17912/micropub.biology.001673

**Published:** 2025-07-18

**Authors:** Ohm H. Patel, Alexandra S. Weisman, Craig P. Hunter

**Affiliations:** 1 Molecular and Cellular Biology, Harvard University, Cambridge, Massachusetts, United States

## Abstract

Gut granules are prominent cytoplasmic organelles that auto fluoresce when excited by much of the visible spectrum confounding fluorescence imaging of endogenous fluorescent reporters. We report a simple, chemical-free fixation method for *C. elegans* that preserves non-cytoplasmic GFP localization for months while nearly eliminating intestinal autofluorescence. To illustrate the utility and limitations of the method, we present representative images of live and heat-fixed worms expressing a variety of membrane and non-membrane localized GFP reporters expressed in diverse tissues, cells, and cellular organelles. We also describe our experience with various experimental parameters. The observed variability of non-membrane-anchored reporters suggests prudent adopters should empirically interrogate the signal fidelity of their specific reporters upon heat fixation.

**Figure 1. Representative images of live or heat-fixed endogenous fluorescent reporters in C. elegans:**
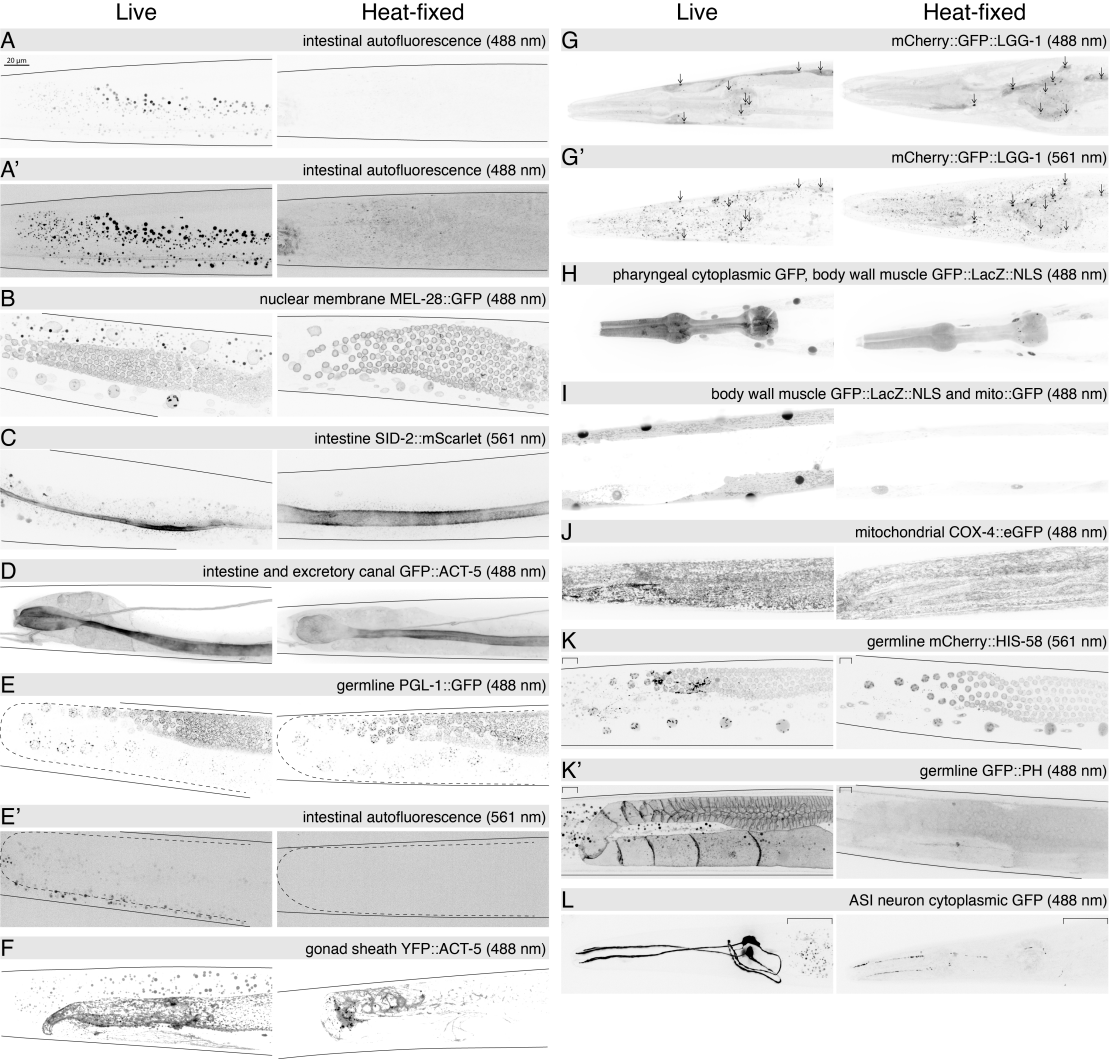
Maximum Intensity Projection (MIP) of spinning disc confocal images (unless otherwise noted) of representative paired live and heat-fixed animals expressing a variety of fluorescent protein reporters. In all images worms are oriented with anterior on the left, ventral down. The image collection and display parameters for each pair of images are identical. All images are at the same scale and resolution. Scale bar in A is 20 µm. Z-stack slice interval was 1 µm, except in I (0.2 µm). Solid lines indicate worm edges, dashed lines outline the gonad arm. Images in panels D, G and L are of Rol worms. Scored sample size(s) of one (n) or two (n, n) independent experiments for live (L) and heat-fixed (HF) conditions are indicated in A-L. n = number of adult hermaphrodite worms imaged within each replicate experiment. A, A') Wild type ( N2 ) showing anterior intestinal (gut granule) autofluorescence (AF) signal. In A) the maximum (non-saturating) signal set point is set to the live image while in A') the maximum set point is set to the heat-fixed sample. L (n = 4, n = 2), HF (n = 6, n = 4). B) GFP:: MEL-28 localization to the nuclear pore, kinetochore, and nucleoplasm in live and heat-fixed mid-body gonad and intestine. Note the lack of 488 nm (green) excitation AF in heat-fixed sample. L (n = 5, n = 6), HF (n = 6, n = 5). C) SID-2 ::mScarlet localization to the intestinal lumen and adjacent endocytic vesicles. Lumenal signal is slightly saturated to better visualize endocytic vesicles. Note the lack of 561 nm (red) excitation AF in heat-fixed sample. The live image shows the thin ribbon-like lumen morphology that is disrupted by heat fixation. L (n = 5, n = 6), HF (n = 6, n = 5). D) GFP:: ACT-5 localization adjacent to intestinal and excretory canal lumenal membrane. Image intensity gamma transformed (0.5). Note the effect of heat fixation on the morphology of the intestinal lumen and the collapse of the excretory canal. L (n = 4), HF (n = 5). E, E') PGL-1 ::GFP localization to germline P granules and excitation AF, 488 nm (green) and 561 nm (red) respectively. Note the lack of 561 nm (red) excitation AF in heat-fixed sample. L (n = 4, n = 7), HF (n = 7, n = 5). F) Gonad expressed YFP:: ACT-5 localizes adjacent to gonad sheath membrane. Heat fixation reduces signal intensity. Image intensity gamma transformed (0.6). Note the lack of 488 nm (green) excitation AF in heat-fixed sample. L (n = 1, n = 5), HF (n = 1, n = 7). G, G') Dual fluorescent mCherry::GFP:: LGG-1 reporter that localizes to autophagosomes (red and green, arrows) and G', autolysosomes (green quenched, red only) in the hypodermal seam cells and pharynx. Heat fixation preserves co-localization. L (n = 4, n = 1), HF (n = 3, n = 1). H) Abundantly expressed cytoplasmic GFP in the pharynx and nuclear localized GFP::LacZ in the body wall muscle are partially quenched by heat fixation. L (n = 4), HF (n = 5). I) Body wall muscle nuclear-localized GFP::LacZ::NLS and mitochondrial-localized mito::GFP are both partially quenched by heat fixation. MIP is approximately one-quarter thickness of worm. L (n = 4), HF (n = 5). J) A single z-section of anterior body region showing inner mitochondrial membrane localized cytochrome c oxidase subunit 4 COX-4 ::eGFP. The live image reveals the highly ordered organization of mitochondria in muscle cells. A similar image plane in the heat-fixed sample shows a similar level of GFP fluorescence but distinct lack of organization. L (n = 6, n = 4), HF (n = 7, n = 5). K, K') Germline expressed nuclear-localized mCherry:: HIS-58 is partially quenched, while germline expressed peripheral membrane GFP::PH (pleckstrin homology domain) is disrupted by heat fixation. Note the lack of 488 nm (green) excitation AF in heat-fixed sample (brackets). L (n = 3, n = 7), HF (n = 4, n = 6). L) Abundantly expressed cytoplasmic GFP in the ASI neuron is disrupted by heat fixation, cell bodies are saturated to allow visualization of neuronal processes. Note the lack of 488 nm (green) excitation AF in heat-fixed sample (brackets). L (n = 9, n = 3*), HF (n = 7, n = 4*). *Replicate acquired on a Zeiss LSM980.

## Description

Tissue fixation is normally achieved by a combination of chemical and environmental conditions that cross-link and or denature proteins with or without extraction of lipids and metabolites (Howat and Wilson, 2014). Here, we show that heat treatment alone can be sufficient to preserve the localization and fluorescence of GFP and mCherry reporters while reducing intestinal autofluorescence (AF). Shown in Figure 1 are examples of various fluorescent protein reporters that are either well or poorly preserved by 95°C fixation (Figure 1). The presence of detectable fluorescence suggests that this brief temperature excursion only partially denatures the fluorescent protein (GFP, YFP, mCherry, mScarlet) or that they refold sufficiently well to be fluorescent. These images also demonstrate that intestinal autofluorescence in both green (488 nm) and red (561 nm) fluorescent channels is significantly reduced. This reduction in AF is most obvious in samples without reporters or where the fluorescent reporter signal is less bright ( Figure 1A- C, E', F, K, K', L). In general, heat fixation preserves the brightness and localization of membrane-bound reporters, including plasma membrane ( Figure 1C ), nuclear membrane ( Figure 1B ), and autophagosomal and autolysosomal membranes ( Figure 1G ). Cytoskeletal ACT-5 protein reporters are also well preserved by heat treatment in the intestine, excretory canal, and germline ( ACT-5 , Figure 1D, F). Cytoplasmic, peripheral membrane, nuclear-localized and mitochondrial-localized protein reporters are poorly preserved ( Figure 1 H-L). Surprisingly, the heat fixation did not disrupt detection of the phase separated liquid condensate P granule marker PGL-1 ::GFP ( Figure 1 E). The heat fixation noticeably altered the morphology of the intestinal lumen from a flattened ribbon to a more symmetrical tube shape ( Figure 1 C, D). The excretory canal also appears to collapse ( Figure 1D ).

Although all images presented in Figure 1 represent same-day fixation and imaging to facilitate direct comparison with the live-imaged samples, fixed animals can be stored at 4°C with addition of sodium azide, to discourage microbial growth, for at least six months with only slightly detectable changes in signal strength. Inclusion of levamisole in the fixation condition can cause morphological artifacts (e.g. elongated nuclei). Most strains were tested in multiple independent replicate experiments ( Figure 1, legend). We have not explored post-heat-fix treatments to increase permeability for addition of non-permeable dyes or antibodies. The heat fixation causes the worms to be near-linear, a benefit for studies that involve measuring worm size.

## Methods

*C. elegans* maintenance as described in (Brenner, 1974). All strains were maintained on normal growth medium plates seeded with OP50 at 20°C.

**Heat Fixation**

Materials: 95°C water, 1.7 ml polypropylene tube, heat block, S. Medium

Procedure:

**Microscopy**

For each strain we mounted and imaged live and heat-fixed day 1 adult hermaphrodite worms prepared on the same day from the same culture. We used levamisole (3 mM) to immobilize live worms for microscopy. Fixed and live worms were mounted on 10% grooved agar pads (Rivera Gomez and Schvarzstein, 2018), trimmed to be smaller than the overlying coverslip, and sealed with beeswax. Identical acquisition parameters (laser power, gain, z-slice intervals) were used for live and heat-fixed animals within each experiment. All displayed images (z-stacks) were collected with a Nikon Spinning Disk Confocal (CSU-W1) microscope using a Plan-Apochromat 60x/1.42 Oil objective, laser lines (filters) 488 nm (525/36 nm) for GFP/YFP and 561 nm (605/52 nm) for mCherry/mScarlet at 1-30% laser power, and slice-intervals of 1.0 or 0.2 µm. In some replicate experiments images were acquired using a LD C-Apochromat 40x/1.1 Water objective on a Zeiss LSM980. Collected images were manipulated (orientation, cropping, brightness and contrast) using FIJI/Image J (version 2.16.0/1.54) (Schindelin et al., 2012). Brightness and contrast display settings were identically adjusted for each pair, images are presented with an unsaturated linear display unless otherwise noted in the figure text. Maximum projection images from z-slices corresponding to the thickness of the worm (or one-quarter thickness in panel I) were processed in FIJI/ImageJ and the figure assembled using Adobe Illustrator.

## Reagents

**Table:** 

**Panel(s)**	**Strain**	**Genotype**	**Available From**	**Reference Images**	**Tissue/Cell Localization of Fluorescent Protein in Adults**	**Organelle Localization**
A, A'	N2	wild-type	Caenorhabditis Genetics Center (CGC)		N/A	N/A
B, C	HC1262	*mel-28* ( *bq5* [GFP:: *mel-28* ]) *sid-2* ( *my95* [ *sid-2* ::mScarlet]) *III*	Hunter Lab	Gómez-Saldivar et al., 2016 ( *bq5* ); Nikonorova et al. 2022 ( *my95* )	All;Intestine	Nuclear membrane, kinetochore and nucleoplasm; Plasma membrane
D	ERT60	*jyIs13* [ *act-5p* ::GFP:: ACT-5 + *rol-6* ( *su1006* )] *II*	CGC	Szumowski et al., 2015	Intestine	Cytoskeleton
E, E'	JH3269	*pgl-1* ( *ax3122* [ *pgl-1* ::GFP]) *IV*	CGC	Putnam et al., 2019	Germline	Cytoplasm (P Granules)
F	WS2170	*opIs110* [ *lim-7p* ::YFP::ACT-5 + *unc-119* (+)] *IV*	CGC	Kinchen et al., 2005	Somatic gonad sheath	Cytoskeleton
G, G'	MAH215	*sqIs11* [ *lgg-1 p* ::mCherry::GFP::LGG-1 + *rol-6* ]	CGC	Chang et al., 2017	Multiple somatic	Autophagosome and Autolysosome
H, I	HC46	*ccIs4251* [(pSAK2) *myo-3p* ::GFP::LacZ::NLS + (pSAK4) *myo-3p* ::mitochondrial GFP + *dpy-20* (+)] *I* ; *mIs11* [ *myo-2p* ::GFP + *pes-10p* ::GFP + F22B7.9 ::GFP] *IV*	Hunter Lab	Winston et al., 2002	Body wall muscle; Pharyngeal muscle	Nucleus,mitochondria and cytoplasm; Cytoplasm
J	JJ2586	*cox-4* ( *zu476* [ *cox-4* ::eGFP::3xFLAG]) *I*	CGC	Raiders et al., 2018	All	Mitochondria (inner membrane)
K, K'	OD95	*ltIs37* [ *pie-1p* ::mCherry:: *his-58* + *unc-119 (* +)] *IV* ; *ltIs38* [ *pie-1p* ::GFP::PH(PLC1delta1) + *unc-119* (+)]	CGC	Essex et al., 2009 ( OD95 ); Raiders et al., 2018 ( OD70 *ltIs44* [ *pie-1p* ::mCherry::PH(PLC1delta1) + *unc-119* (+)] *V* adult hermaphrodite gonad)	Germline	Nucleus; Plasma Membrane
L	FK181	*ksIs2* [ *daf-7p* ::GFP + *rol-6* ( *su1006* )]	CGC	Murakami et al., 2001	ASI neuron	Cytoplasm
